# Prophylactic platelet transfusion response in critically ill patients: a prospective multicentre observational study

**DOI:** 10.1186/s13054-023-04650-z

**Published:** 2023-09-27

**Authors:** Florian Reizine, Sarah Le Marec, Anthony Le Meur, Maëlys Consigny, Florian Berteau, Laetitia Bodenes, Marie Geslain, Zoe McQuilten, Catherine Le Niger, Julien Huntzinger, Philippe Seguin, Jean-Baptiste Thibert, David Simon, Jean Reignier, Pierre-Yves Egreteau, Jean-Marc Tadié, Olivier Huet, Pierre Asfar, Stephan Ehrmann, Cécile Aubron

**Affiliations:** 1 Maladies Infectieuses Et Réanimation Médicale, CHU de Rennes, Rennes, France; 2 Service de Réanimation Polyvalente, CH de Vannes, Vannes, France; 3 Service de Médecine Intensive Réanimation, Université de Bretagne Occidentale, Centre Hospitalo-Universitaire de Brest, Site La Cavale Blanche, Bvd Tanguy Prigent, 29609 Brest Cedex, France; 4 Service de Médecine Intensive Réanimation, CHU de Nantes, Nantes, France; 5 Service de Biostatistiques, CHU de Brest, Brest, France; 6 Service de Réanimation Polyvalente, CH de Morlaix, Morlaix, France; 7 Département d’anesthésie-Réanimation, Université de Bretagne Occidentale, CHU de Brest, Brest, France; 8 Australian and New Zealand Intensive Care Research Centre (ANZIC-RC), Monash University, Melbourne, Australia; 9 Unité d’hémovigilance, CHU de Brest, Brest, France; 10 Service de Réanimation Chirurgicale, CHU de Rennes, Rennes, France; 11 Etablissement Français du Sang Bretagne, Rennes, France; 12 Service de Médecine Intensive Réanimation, CHU d’Angers, Angers, France; 13 Médecine Intensive Réanimation, CHRU de Tours INSERM CIC 1415, CRICS-TriggerSEP F-CRIN Research Network, INSERM U1100, Université de Tours FR, Tours, France

**Keywords:** Platelet transfusion response, Prophylactic transfusion, Critically ill patient, Bleeding

## Abstract

**Background:**

Response to prophylactic platelet transfusion is suspected to be inconsistent in critically ill patients questioning how to optimize transfusion practices. This study aimed to describe prophylactic platelet transfusion response, to identify factors associated with a suboptimal response, to analyse the correlation between corrected count increment and platelet count increment and to determine the association between poor platelet transfusion response and clinical outcomes.

**Methods:**

This prospective multicentre observational study recruited patients who received at least one prophylactic platelet transfusion in one of the nine participating intensive care units for a period up to 16 months. Poor platelet transfusion response was defined as a corrected count increment (CCI) that adjusts for platelet dose and body surface area, less than 7 at 18–24 h after platelet transfusion. Factors associated with poor platelet transfusion response were assessed in a mixed-effect model. Sensitivity analyses were conducted in patients with and without haematology malignancy and chemotherapy.

**Results:**

Poor platelet transfusion response occurred in 349 of the 472 (73.9%) prophylactic platelet transfusions and in 141/181 (77.9%) patients. The mixed-effect model identified haemoglobin at ICU admission (odds ratio (OR): 0.79 [95% confidence interval (CI) 0.7–0.89]) and body mass index (BMI) (OR: 0.93 [0.89–0.98]) being positively and independently associated with platelet transfusion response, while a haematological malignancy (OR 1.93 [1.09–3.43]), sepsis as primary ICU admission diagnosis (OR: 2.81 [1.57–5.03]), SOFA score (OR 1.10 [1.03; 1.17]) and maximum storage duration of platelet (OR: 1.24 [1.02–1.52]) were independently associated with a suboptimal platelet increment. Clinical outcomes did not differ between groups, nor the requirement for red blood cells. Poor platelet transfusion response was found in 93.5% of patients with haematology malignancy and chemotherapy.

**Conclusions:**

In this study of critically ill patients, of whom more than half had bone marrow failure, almost three quarters of prophylactic platelet transfusions led to suboptimal platelet increment measured 18 to 24 h following platelet transfusion. Platelet storage duration was the only factor associated with poor platelet response that may be accessible to intervention.

*Trial registration* in October 2017: ClinicalTrials.gov: NCT03325140.

**Supplementary Information:**

The online version contains supplementary material available at 10.1186/s13054-023-04650-z.

## Background

Thrombocytopenia is frequent in critically ill patients and is associated with an increased risk of bleeding complications [1]. As a consequence, platelet transfusions are given to thrombocytopenic patients to prevent spontaneous bleeding or bleeding related to invasive procedures. There is no robust evidence to support clinical practices of prophylactic platelet transfusion in critically ill patients. Randomized clinical trials investigating prophylactic platelet transfusions are few and have mostly been performed in haematology patients. One trial conducted in haematology cancer patients reported a decrease in bleeding rate in patients transfused to maintain platelet count above 10 × 10^9^/L compared to no platelet transfusion [2]. In another trial, also conducted in patients with haematological malignancies, a strategy of prophylactic platelet transfusion compared to therapeutic transfusion (only administered when bleeding) was associated with reduced bleeding (World Health Organization [WHO] grade 2, 3 or 4 bleeding), although similar rates of bleeding were observed in the subgroup of patients receiving autologous stem-cell transplantation [3]. Although van Baarle et al., recently reported a decreased risk of bleeding after central venous catheter placement in patients with thrombocytopenia between 10 and 50 × 10^9^/L who received platelet transfusion compared to patients without platelet transfusion, current guidelines are mainly based on poor quality evidence or evidence from the onco-haematological population [4, 5]. In thrombocytopenic non-bleeding critically ill patients, the platelet count threshold for platelet transfusion might be higher than in other patient populations because of a higher bleeding risk and also because these patients often suffer from underlying sepsis, fever or infection, factors that might jeopardize the platelet transfusion response [5, 6]. On the other hand, platelet transfusion is associated with adverse events, making it crucial to limit their use to the indications for which the benefits outweigh the risks.

Poor platelet transfusion response has been reported in critically ill patients [7, 8] [9]. However, studies that have analysed determinants of platelet transfusion response in this patient group often focused on cancer patients [7], were retrospective [8] or single centre [9], questioning the generalisability of their results. The methods to analyse platelet transfusion response vary between studies and might also be sources of biases, as they often do not adjust for platelet dose or body surface area, which are both important confounders. In addition, the association between platelet transfusion response and patients’ outcomes remains unknown.

We therefore conducted a prospective multicentre observational study that aimed to: (1) describe prophylactic platelet transfusion response at 18–24 h and the factors associated with poor platelet transfusion response as defined by the corrected count increment (CCI); (2) determine the platelet count increment that is predictive of a CCI less than 7; and (3) investigate whether suboptimal platelet transfusion response was associated with clinical outcomes including mortality and bleeding events.

## Methods

### Study design and setting

We conducted a prospective multicentre observational study from June 2018 and up to November 2019 in nine French intensive care units (ICUs) of seven hospitals (five university affiliated hospital and two community hospitals). Two ICUs were surgical, five were medical and two were general ICUs. The bed capacity in the ICUs ranged from 12 to 28 beds.

Patients older than 18 years who received at least one prophylactic platelet transfusion in intensive care were eligible for enrolment. Prophylactic platelet transfusion was defined as: (1) platelet transfusion given to patients with thrombocytopenia without bleeding (defined as bleeding of WHO grades 2–4) or (2) platelet transfusion given prior to an invasive procedure in patients with thrombocytopenia. Platelet transfusions were given in accordance with the French national guidelines (prophylactic platelet transfusion in the absence of surgery or invasive procedure was given to maintain platelet count above 10 to 20 × 10^9^/L, platelet transfusion was given to reach a platelet count above 100 × 10^9^/L prior to neurosurgery, or above 50 × 10^9^/L prior to general surgery, lumbar puncture or central intravascular catheter insertion) [6]. Prophylactic platelet transfusions related to an invasive procedure or surgery were defined as only those that were administered prior to the invasive procedure and/or the surgery.

The study protocol was approved by The Comité de Protection des Personnes, Tours-Ouest 1 (approval number 2017T3-22). The research was conducted in accordance with the ethical standards of this responsible committee on human experimentation and with the Helsinki Declaration of 1975. Patients, or their next of kin when they were unable to consent, received oral and written information about the study and consent was obtained before inclusion. Then, the patient was informed of the study and express consent was obtained as soon as possible. The study was registered on the ClinicalTrials.gov website (NCT03325140).

### Data collection

Demographic characteristics including age, gender, body mass index (BMI), ABO blood group and patients’ comorbidities with haematological malignancy, immunosuppression, cirrhosis and chronic kidney disease were recorded. The severity of illness and organ failures at ICU admission were assessed using the Simplified Acute Physiology Score II (SAPS II) [10] and the Sequential Organ Failure Assessment (SOFA) score [11]. Haemoglobin concentration, platelet count and prothrombin time at ICU admission were also collected.

Recorded clinical and biological parameters before or on the day of platelet transfusion included heart rate, body temperature, systolic arterial blood pressure and mean arterial blood pressure.

Data related to platelet concentrates were retrieved from the French Blood Banks (Etablissement Français du Sang) of each hospital and included duration of platelet storage, platelet dose (number of platelets administered), preparation from pooled or single donors and platelet ABO compatibility.

### Platelet concentrates

Two different kinds of platelet concentrates, pooled whole-blood-derived platelets and apheresis platelets, were given based on their availability. The pooled whole-blood-derived platelets are made up of different whole-blood donations (8 usually, maximum 12) of the same ABO blood group. The apheresis platelets are collected in a licensed device and stored in an additive/substitutive storage solution. Both were treated for pathogen reduction (Amotosalen and UVA), since November 2017. Platelet concentrates were all leucodepleted. Each unit contained at least 2 × 10^11^ platelets and less than 10^6^ leucocytes. The platelet concentrates were stored at a temperature between + 20 °C and + 24 °C with slow and continuous agitation. Their shelf life was 7 days from the date and time of the collection. The number of platelets transfused was calculated on the basis of patient weight according to the following formula 0.5 to 0.7 × 10^11^ platelets per 10 kg of weight. The volume of platelet unit varied between 200 and 400 ml for apheresis platelets and between 200 and 300 ml for pooled whole-blood-derived platelets.

### Platelet transfusion response and outcomes

A corrected count increment (CCI) calculated by the following formula: [(post-transfusion platelet count)—(pre-transfusion platelet count)] x (body surface area) / (number of platelets transfused) < 7 at 18–24 h after platelet transfusion was used to define poor platelet transfusion response [6, 7]. The CCI is an internationally accepted method to measure platelet increment following transfusion, using the platelet dose administered irrespective of how the dose was prescribed. Moreover, as platelet transfusion response is easier assessed by analysing platelet count after platelet transfusion, we compared the platelet count increment (defined as post-transfusion platelet count—pre-transfusion platelet count) with the CCI. Clinical outcomes included 28-day mortality, occurrence of bleeding within the 24 h following platelet transfusion, ICU length of stay and duration of mechanical ventilation. Bleeding events of WHO grades 2–4 (with bleeding of grades 3 and 4 considered as major bleeding) were recorded [12]. Requirement of blood components including fresh frozen plasma (FFP) and red blood cells (RBC) as a surrogate of major bleeding after platelet transfusion were also collected.

### Statistical analysis

Continuous variables are reported as median (interquartile ranges (IQR)) and categorical variables as proportions (%). Characteristics of patients with at least one poor platelet transfusion response were compared to those of patients without poor platelet transfusion response. Characteristics of platelet transfusion with and without suboptimal platelet increment were compared. The Mann–Whitney test was used for quantitative data and the Chi-square or Fisher tests for qualitative data, as appropriate. Survival curves up to day 28 of patients with and without poor platelet transfusion response were established by the Kaplan–Meier method and compared using the log-rank test. Multiple imputation methods were used in the case of missing data. All missing data were imputed using SAS Fully Conditional Specification (FCS) multiple imputation with the SAS MI procedure. This method is based on an iterative algorithm (20 iterations in this study).

Each iteration provides a data set whose imputed missing values are based on plausible values representing the uncertainty about the correct value to impute, obtained by using a linear regression to impute missing values for a continuous variable, a logistic regression to impute missing values for a binary variable and a discriminant method function to impute missing values for a categorical variable.

We used 10 imputed data sets. Estimates from each imputed data sets were then pooled to generate a single set of estimates with the SAS MIANALYZE procedure [13]. We used a multivariable generalized linear mixed-effects model (GLM), to account for repeated measures within individual patients, to assess the association between patient and platelet characteristics with platelet transfusion response. This GLM model was constructed with a logit link, a distribution according to a binary distribution, a random effect for participant to account for repeated transfusions, and the other variables as fixed effects. The variables included in the GLM model were determined by their potential impact on the efficacy of platelet transfusion. The patient-related variables included age, gender, BMI, pregnancy, comorbidities, SAPS II, past history of platelet transfusion, type of admission, sepsis as the primary ICU admission diagnosis, platelet count, haemoglobin and prothrombin time (PT) at ICU admission. Transfusion-related variables included heart rate, temperature, systolic arterial pressure, therapeutic anticoagulation, SOFA on the day of platelet transfusion and maximal platelet storage duration. The results of these analyses are presented as odds ratio (OR) with their 95% confidence interval (95% CI).

Although CCI is the reference method to determine platelet transfusion response, its use at the bedside is limited as it is based on the transfused platelet dose and the body surface area. Therefore, we analysed the correlation between platelet count increment and CCI calculated between 18 and 24 h after platelet transfusion. Comparison between platelet transfusion response based on the CCI and platelet count increments was done by performing receiver operating characteristic (ROC) curves.

As the reason for thrombocytopenia might impact on platelet transfusion response, subgroup analyses were conducted in patients with and without bone marrow failure, which was defined as a haematology malignancy with chemotherapy.

Two-sided tests were performed and considered to reach statistical significance when the p-value was < 0.05. Analyses were done using SAS software version 9.4.

## Results

### Study population

**Fig. 1:**
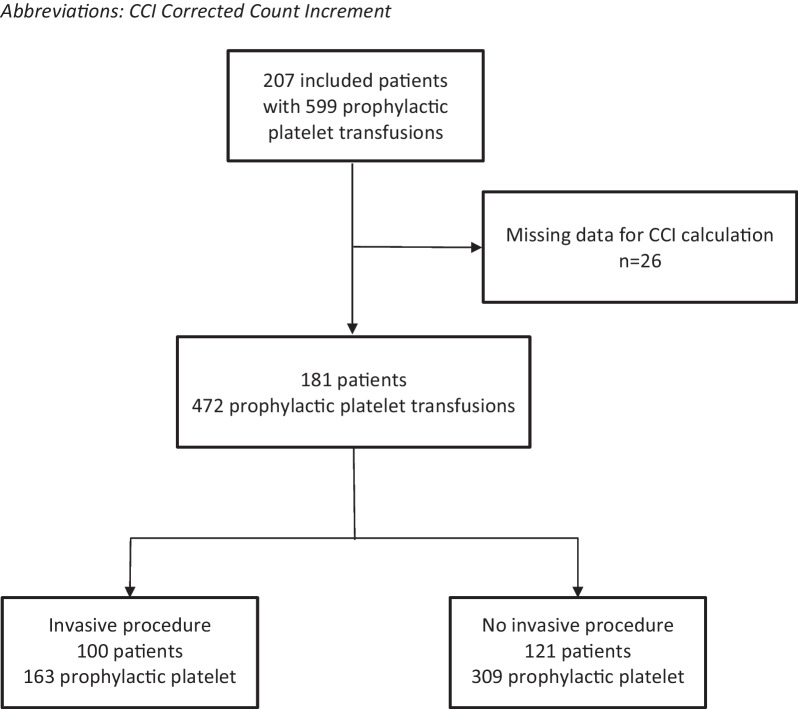
Flowchart of the study. *CCI* Corrected count increment

Over the study period, of the 571 patients who received at least one platelet transfusion in ICU, 222 (39%) could not be included because consent could not be obtained (refusal and/or death). Among the other 349 patients, 142 received only therapeutic platelet transfusions (i.e. transfusion given to patients with bleeding) and 207 patients received at least one prophylactic platelet transfusion. Complete data to calculate the CCI were missing in 26 patients, leading to the analysis of platelet transfusion efficacy in 181 patients receiving 472 prophylactic platelet transfusion episodes. Of the 472 platelet transfusions, 163 (34.5%) were given to 100 patients prior to an invasive procedure and 309 (65.5%) were given to 121 patients with thrombocytopenia not undergoing an invasive procedure or surgery (Fig. 1). For patients undergoing a procedure, surgery was the most frequent procedures, accounting for 52 platelet transfusions (31.9%) (Additional file 1: Table S1).

Baseline characteristics of the 181 patients are displayed in Table 1. They were mainly male (56.9%) with a median age of 60 years [48–68]. Overall, 96 (53.0%) patients had a haematological malignancy, and these patients received the majority of platelet transfusions (268/472; 56.8%). Sepsis was the most common primary ICU admission diagnosis, reported in 66 patients (36.5%). The median SAPS II score was 52 [40–65]. Median platelet count at ICU admission was 34 × 10^9^ /L [16 × 10^9^–70 × 10^9^].

**Table 1:** Baseline characteristics of patients with and without at least one platelet transfusion with poor response

Variables	All patients *n* = 181		Patients with at least one PT with poor response *n* = 141	Patients without poor PT response *n* = 40	*P* value
*Demographic characteristics*					
Age, years	60 (48–68)		60 (46–67)	66 (56–73)	0.0109
Male sex	103 (56.9%)		77 (54.6%)	26 (65.0%)	0.2415
Pregnancy^a^	55 (33.1%)		46 (35.4%)	9 (25.0%)	0.2414
BMI	24.9 (22.7–28.4)		25.0 (22.4–28.4)	24.8 (23.2–28.1)	0.5492
History of platelet transfusion^b^	76 (63.9%)		64 (65.3%)	12 (57.1%)	0.4798
Cardiovascular disease	48 (26.5%)		35 (24.8%)	13 (32.5%)	0.3316
Diabetes mellitus	21 (11.6%)		15 (10.6%)	6 (15.0%)	0.4157
Haematological malignancy	96 (53.0%)		85 (60.3%)	11 (27.5%)	0.0002
Solid neoplasia	32 (17.7%)		26 (18.4%)	6 (15.0%)	0.6147
Chemotherapy	93 (51.4%)		85 (60.3%)	8 (20.0%)	< 0.0001
Chronic respiratory disease	18 (9.9%)		14 (9.9%)	4 (10.0%)	1.0000
Chronic kidney injury	7 (3.9%)		3 (2.1%)	4 (10.0%)	0.0435
Anti-platelet agents	24 (13.3%)		16 (11.3%)	8 (20.0%)	0.1544
*Type of admission*					
Post-operative admission	32 (15.4%)		22 (13.3%)	10 (23.8%)	
Medical admission	176 (84.6%)		144 (86.7%)	32 (76.2%)	
Post-operative admission	23 (12.7%)		14 (9.9%)	9 (22.5%)	0.0351
Primary ICU admission diagnosis sepsis	66 (36.5%)		59 (41.8%)	7 (17.5%)	0.0048
Diagnosis at ICU admission					0.0109
Respiratory distress	29 (16.0%)		24 (17.0%)	5 (12.5%)	
Trauma with brain injury	3 (1.7%)		0 (0.0%)	3 (7.5%)	
Trauma without brain injury	1 (0.6%)		1 (0.7%)	0 (0.0%)	
Neurologic	11 (6.1%)		8 (5.7%)	3 (7.5%)	
Acute kidney injury	1 (0.6%)		1 (0.7%)	0 (0.0%)	
Cardiovascular	8 (4.4%)		5 (3.5%)	3 (7.5%)	
Haematologic	15 (8.3%)		12 (8.5%)	3 (7.5%)	
Uro-digestive	3 (1.7%)		2 (1.4%)	1 (2.5%)	
Haemorrhage	12 (6.6%)		7 (5.0%)	5 (12.5%)	
Metabolic	3 (1.7%)		2 (1.4%)	1 (2.5%)	
Other	29 (16.0%)		20 (14.2%)	9 (22.5%)	
*Patient ABO Group*^*c*^					
A	73 (42.7%)		60 (44.4%)	13 (36.1%)	0.5711
O	73 (42.7%)		54 (40.0%)	19 (52.8%)	
B	21 (12.3%)		17 (12.6%)	4 (11.1%)	
AB	4 (2.3%)		4 (3.0%)	0 (0.0%)	
Rhesus positive^d^	150 (87.7%)		120 (88.2%)	30 (85.7%)	0.7727
*Clinical and laboratory findings at ICU admission*					
SAPS II^e^	52 (40–65)		52 (41–65)	51 (37–63)	0.3854
Platelet count, × 10^9^/L	34 (16–70)		27 (12–59)	58 (34–143)	0.0001
Haemoglobin, g/dl	8.8 (7.5–10.5)		8.5 (7.4–9.9)	11.3 (8.7–13.4)	< 0.0001
Prothrombin time < 70%^f^	122 (70.9%)		97 (73.5%)	25 (62.5%)	0.1801
*Clinical outcomes*					
Number of RBC units transfused	0 (0–3)		0 (0–3)	0 (0–3)	0.5151
ICU LOS, days	11 (5–21)		10 (5–21)	11 (4–19)	0.9632
Hospital LOS, days	24 (12–45)		24 (12–47)	24 (11–36)	0.3293
MV duration, days	4 (0–11)		3 (0–11)	5 (0–12)	0.5245
Survival at ICU discharge	133 (73.5%)	104 (73.8%)		29 (72.5%)	0.8735

^a^Missing data: *n* = 15

^b^Missing data: *n* = 62

^c^Missing data: *n* = 10

^d^Missing data: *n* = 10

^e^Missing data: *n* = 1

^f^data: *n* = 9

Data are presented as median (IQR: interquartile) or n (%)

*BMI* Body mass index, *ICU* intensive care unit, *LOS* length of stay, *RBC* red blood cells, *PT* platelet transfusion, *SAPS II* Simplified Acute Physiology Score II, *MV* mechanical ventilation

### Patient-related characteristics and platelet transfusion response

The median number of platelets that were transfused per episode was 3.7 × 10^11^ platelets [3.3–4.8] and the median number of platelet concentrates transfused for each episode of platelet transfusion was 1 [1]. The mean CCI was 4.8 (standard deviation 10.0). 77.9 per cent of patients (141/181) had at least one platelet transfusion with a poor response based on the CCI. Parameters associated with poor platelet transfusion response were a lower platelet count at ICU admission (27 × 10^9^/L [12 × 10^9^—59 × 10^9^] versus 58 × 10^9^/L [34 × 10^9^–143 × 10^9^]; p = 0.0001), a lower ICU admission haemoglobin (8.5 g/dL ​​[7.4–9.9] versus 11.3 g/dL ​​[8.7–13.4]; p < 0.0001), a haematological malignancy (60.3% of patients with haematological malignancy in patients with poor platelet transfusion response versus 27.5% in patients without a poor transfusion response, p < 0.0001) and sepsis as primary ICU admission diagnosis (Table 1).

### Platelet transfusion-related features and poor platelet transfusion response

Of the 472 prophylactic platelet transfusions, 349 (73.9%) fulfilled the poor platelet transfusion response criteria. Clinical features prior to platelet transfusion that were associated with a CCI less than 7 are presented in Table 2. A higher heart rate (106 bpm [93–120] versus 98 bpm [85–110]; *p* = 0.0001) was associated with poor platelet transfusion response. When considering platelet characteristics, ABO compatibility did not affect the CCI, while patients with poor platelet transfusion response had higher maximum platelet storage duration (5 days [4, 5] versus 4 days [3–5]; p = 0.0152).

**Table 2:** Comparison of characteristics of platelet transfusion with and without suboptimal response based on a CCI < 7

Variables	All episodes *n* = 472		Suboptimal PT response *n* = 349	Optimal PT response *n* = 123	*P* value
*Clinical and biological features before transfusion*					
Heart rate (/min)	104 (90–119)		106 (93–120)	98 (85–110)	0.0001
Heart rate > 100/min	282 (59.7%)		224 (64.2%)	58 (47.2%)	0.0009
Temperature, °C^a^	37.0 (36.4–37.7)		37.1 (36.5–37.7)	36.9 (36.3–37.5)	0.0527
Temperature > 39°C^a^	20 (4.4%)		18 (5.3%)	2 (1.7%)	0.0920
Mean arterial BP, mmHg	76.0 (69–88)		76 (69–88)	77 (71–85)	0.8933
Therapeutic anticoagulation	50 (10.6%)		28 (8.0%)	22 (17.9%)	0.0022
AKI requiring RRT	97 (20.6%)		64 (18.3%)	33 (26.8%)	0.0451
ECMO	8 (1.7%)		6 (1.7%)	2 (1.6%)	1.0000
Infection requiring antibiotics	397 (84.1%)		298 (85.4%)	99 (80.5%)	0.2013
SOFA score^b^	8 (6–13)		9.0 (6–13)	8.0 (6.0–12)	0.0267
Platelet count, × 10^11^/L	15 (9–27)		14 (8–24)	20 (12–36)	< 0.0001
*Transfusion-related characteristics*					
*Transfusion Indication*					0.0602
Prophylactic without invasive procedure	163 (34.5%)		112 (32.1%)	51 (41.5%)	
Prophylactic prior Invasive procedure	309 (65.5%)		237 (67.9%)	72 (58.5%)	
Platelet dose, × 10^11^	3.7 (3.3–4.8)		3.7 (3.3–4.7)	3.7 (3.3–4.8)	0.9069
Apheresis platelet concentrate	155 (32.8%)		119 (34.1%)	36 (29.3%)	0.3268
Pooled platelet concentrate	317 (67.2%)		230 (65.9%)	87 (70.7%)	
ABO compatibility^c^	347 (77.6%)		256 (76.9%)	91 (79.8%)	0.5145
Maximum platelet storage duration, days	4 (3–5)		5 (4–5)	4 (3–5)	0.0152
Maximum of storage time, > 4days	224 (47.5%)		177 (50.7%)	47 (38.2%)	0.0169
24-h fluid balance, mL^d^	825 (− 373–1926)		800 (− 482–1874)	987 (− 200–2000)	0.7883

^a^Missing data: *n* = 14

^b^Missing data: *n* = 23

^c^Missing data: *n* = 25

^d^Missing data: *n* = 13

Data are presented as median (IQR: interquartile), n (%). P values comparing patients are tested by Mann–Whitney (continuous variables) and Chi2 or Fisher tests (categorical variables)

*AKI* acute kidney injury, *BP* blood pressure, *ECMO* extracorporeal membrane oxygenation, *RRT* renal replacement therapy, *SOFA* Sequential Organ Failure Assessment

### Parameters independently associated with poor platelet transfusion response

**Fig. 2:**
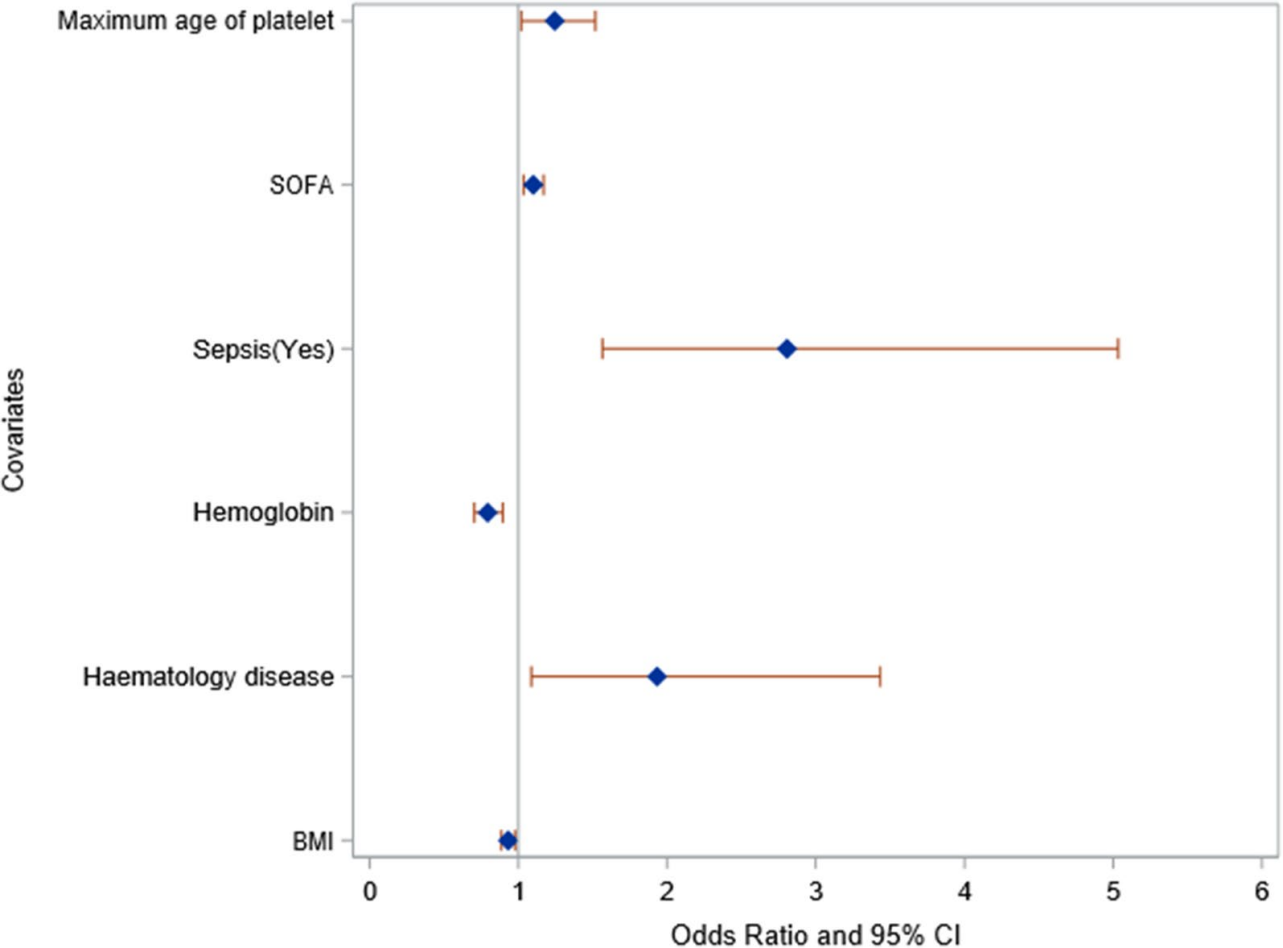
Forest plot of multivariate mixed-effect model to evaluate the association between demographic and transfusion-related characteristics with platelet transfusion response. *BMI* body mass index, *SOFA* Sequential Organ Failure Assessment

The mixed-effect model identified BMI (odds ratio (OR): 0.93 [95% confidence interval (CI) 0.89; 0.98]; *p* = 0.0041) and ICU admission haemoglobin (OR: 0.79 ​​[0.7;0.89]; *p* = 0.0001) to be independently and positively associated with platelet transfusion response, while haematological malignancy (OR: 1.93 ​​[1.09;3.43]; *p* = 0.0246), sepsis as primary ICU admission diagnosis (OR: 2.81 [1.57; 5.03]; *p* = 0.0005), the SOFA score at time of platelet transfusion (OR: 1.10 [1.03; 1.17]; *p* = 0.0022) and the maximum age of platelet unit (OR: 1.24 [1.02; 1.52]; *p* = 0.0315) were independently associated with poor platelet transfusion response, based on the CCI (Table 3 and Fig. 2).

**Table 3:** Univariate and multivariate mixed-effect model to evaluate association between demographic and transfusion-related characteristics with poor platelet transfusion response

Variables	Univariate analysis			Multivariate analysis		
	OR	95% CI	*P* value	OR	95% CI	*P* value
*Baseline characteristics*						
Age (years)	0.98	[0.97–1.00]	0.0812			
Gender (ref: male sex)	1.27	[0.75–2.14]	0.3682			
BMI	0.96	[0.92–1.00]	0.0743	0.93	[0.89; 0.98]	0.0041
Pregnancy	1.39	[0.81–2.38]	0.2280			
Haematological malignancy	1.90	[1.13–3.18]	0.0157	1.93	[1.09–3.43]	0.0246
Cardiovascular disease	1.00	[0.54–1.84]	0.9927			
Chronic kidney injury	0.22	[0.05–0.94]	0.0409			
SAPS II	1.00	[0.99–1.02]	0.6690			
Antecedent of platelet transfusion (ref = "No")	0.90	[0.49–1.65]	0.7362			
Platelet count at ICU admission	0.99	[0.99–1.00]	0.0548			
Haemoglobin	0.79	[0.71–0.89]	< 0.0001	0.79	[0.7–0.89]	0.0001
PT (ref = " < 70%")	0.72	[0.41–1.26]	0.2484			
Type of admission (ref = post-operative)	2.69	[1.25–5.79]	0.0118			
Primary ICU admission diagnosis sepsis (ref: No)	2.91	[1.67–5.07]	0.0002	2.81	[1.57–5.03]	0.0005
*Transfusion episode-related features*						
Heart rate before transfusion > 100/min	1.93	[1.23–3.03]	0.0041			
Temperature before transfusion (ref < 39°C)	3.71	[0.8–17.3]	0.0946			
Infection requiring antibiotics	1.40	[0.78–2.54]	0.2613			
Systolic arterial blood pressure before transfusion	1.00	[0.99–1.01]	0.6427			
Therapeutic anticoagulation	0.45	[0.22–0.89]	0.0219			
SOFA score	1.05	[0.99–1.11]	0.0859	1.10	[1.03–1.17]	0.0022
24-h fluid balance	1.00	[1.00–1.00]	0.6944			
Maximum platelet storage duration	1.27	[1.05–1.54]	0.0143	1.24	[1.02–1.52]	0.0315

The corrected count increment was treated as a binary variable (upper than 7 or not).

*BMI* body mass index, *PT* prothrombin time, *SAPS II* Simplified Acute Physiology Score II, *SOFA* Sequential Organ Failure Assessment

### Platelet count increment and CCI

Platelet count increment was correlated with the CCI and yielded a combined area under the ROC curve of 0.9906 (Additional file 1: Fig. S1). The platelet count increment value that best correlated with a CCI > 7 was 15 × 10^9^/L.

### Clinical outcomes

**Fig. 3:**
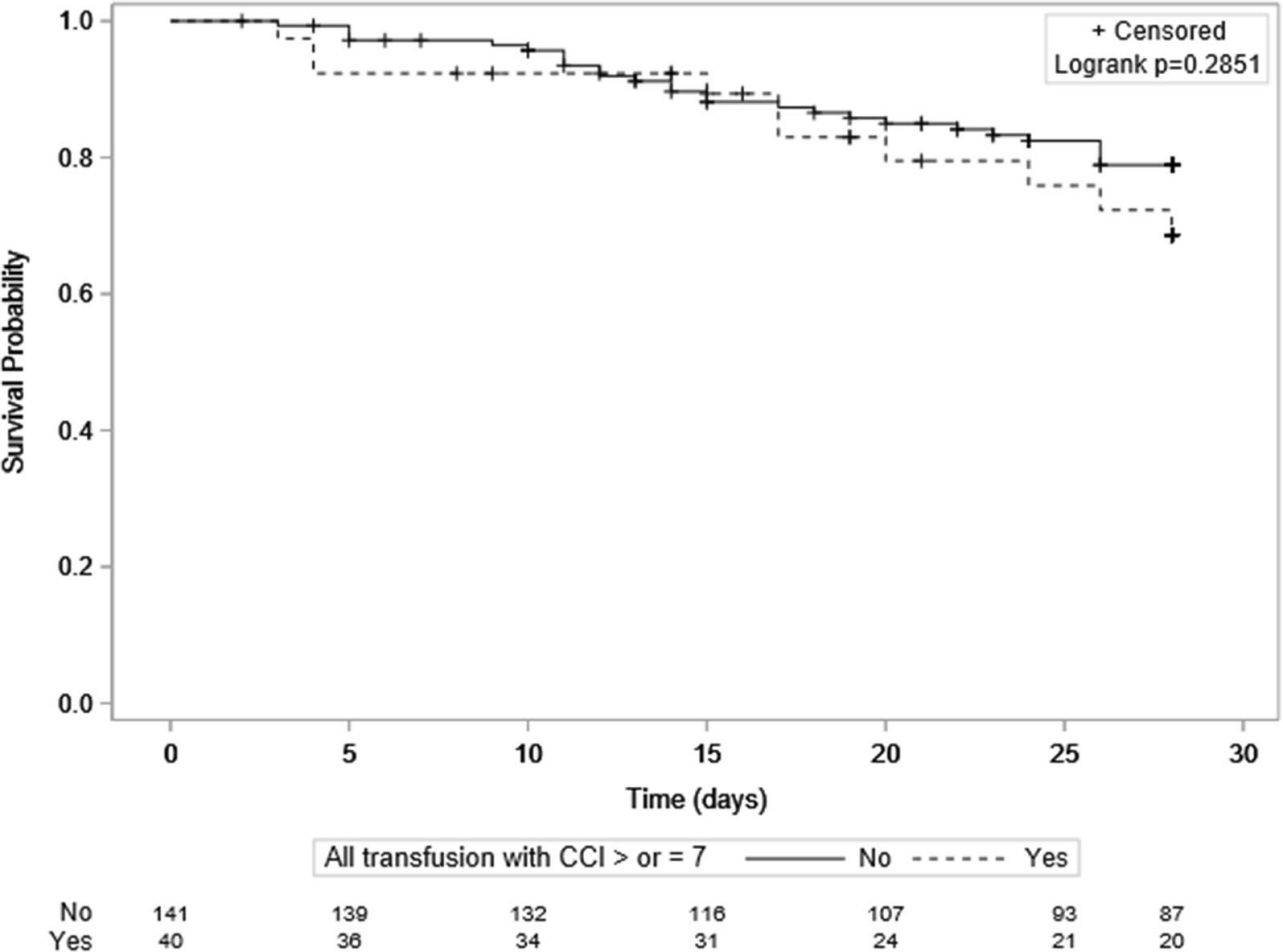
Survival until day 28 according to platelet transfusion response (p-value log rank = 0.2851). *CCI* Corrected count increment

There was no difference in clinical outcomes between patients with and without poor platelet transfusion response (Tables 1 and 4). Patients with and without poor platelet transfusion response had a similar 28-day survival (log-rank test: *p* = 0.2851) (Fig. 3), a similar mechanical ventilation duration, as well as ICU length of stay (Table 1). The requirement for FFP or RBC within the 24 h after platelet transfusion was the same irrespective of the platelet transfusion response (Table 4).

**Table 4:** Bleeding events and RBC and FFP transfusion requirement within the 24 h after platelet transfusion according to platelet transfusion response

Variables	All episodes *n* = 472		Suboptimal PT response *n* = 349	Optimal PT response *n* = 123	*P* value
*WHO grade 2 to 4 bleeding*					
Indication for transfusion: Thrombocytopenia with surgery/invasive procedure (*n* = 163)	29 (17.8%)		17 (15.2%)	12 (23.5%)	0.2688
Thrombocytopenia without surgery/invasive procedure (*n* = 309)	38 (12.3%)		32 (13.5%)	6 (8.3%)	0.2422
*WHO grade 3 or 4 bleeding*					
Indication for platelet transfusion: Thrombocytopenia with surgery or invasive procedure (*n* = 29)	11 (37.9%)		5 (29.4%)	6 (50.0%)	0.4384
Thrombocytopenia without surgery or invasive procedure (*n* = 38)	27 (71.0%)		24 (75%)	3 (50%)	0.3287
*Transfusions within the 24 h after platelet transfusion*					
RBC units	0 (0–8)		0 (0–8)	1 (0–7)	0.7284
Requirement of at least one RBC unit	220 (48.0%)		159 (47.0%)	61 (50.8%)	0.4197
Requirement of at least one FFP	60 (13.1%)		43 (12.7%)	17 (14.2%)	0.6870

Data are presented as median (IQR: interquartile), n (%). P values comparing patients are tested by Mann–Whitney (continuous variables) and Chi2 or Fisher tests (categorical variables)

*FFP* Fresh Frozen Plasma, *PT* Platelet Transfusion, *RBC* Red Blood Cells, *WHO* World Health Organization

### Sensitivity analyses

Of the 181 patients analysed, 77 patients had a haematological malignancy and received chemotherapy. These patients received the majority of platelet transfusions (268/472; 56.8%). Platelet response was particularly low in these patients, with 93.5% (72/77) receiving at least one platelet transfusion with a poor transfusion response at 18–24 h. None of the baseline parameters assessed differed between haematology patients with or without poor platelet transfusion response (Additional file 1: Table S2). When analysing the characteristics of platelet transfusion according to transfusion response, the heart rate before transfusion was higher in transfusion with suboptimal transfusion response (106 [95–120] versus 100 [90–114]; *p* = 0.0479), as well as the SOFA score (Additional file 1: Table S3). The mixed-effect model identified sepsis as primary ICU diagnosis (OR: 2.43 [1.05; 5.64]; *p* = 0.0390) and SOFA score at the time of platelet transfusion to be independently associated with poor platelet transfusion response (Additional file 1: Table S4). Finally, suboptimal platelet transfusion was associated with a longer ICU length of stay, but not with other clinical outcomes (Additional file 1: Table S5).

The analysis of the study population excluding patients with haematological malignancy and chemotherapy (*n* = 104) found 66.3% of patients with at least one platelet transfusion with a CCI lower than 7, and similar differences in baseline characteristics and transfusion-related features according to platelet transfusion response as the overall population (Additional file 1: Tables S6–S8).

## Discussion

In this prospective, multicentre observational study, 73.9% of prophylactic platelet transfusions had a poor response with a CCI less than 7 between 18 and 24 h after platelet administration. The factors independently associated with poor platelet transfusion response included underlying haematological malignancy, sepsis as primary diagnosis, the SOFA score and the maximum duration of platelet storage, while the ICU admission haemoglobin and higher BMI were positively associated with platelet transfusion response. In this heterogeneous population of critically ill patients, patients with poor platelet transfusion response had similar clinical outcomes, including mortality and RBC requirements than patients without suboptimal platelet increment. Patients with haematology malignancy and chemotherapy, who accounted for more than half of the study population, experienced high rate of poor platelet transfusion responses. In this subgroup analysis, sepsis as primary diagnosis was also the parameter the most strongly associated with poor platelet transfusion response.

Poor platelet transfusion response has been previously reported in critically ill patients [7–9, 14]. Baron et al., in a retrospective single-centre study, reported a 54.6% rate of poor platelet transfusion response based on similar criteria as ours [7]. However, this study included only cancer patients with hypoproliferative thrombocytopenia. The authors analysed both therapeutic and prophylactic transfusions [7] together, and the CCI is unlikely to be a relevant criterion to determine efficacy of platelet transfusion in actively bleeding patients. Arnold et al., in a retrospective single centre study, reported a rate of 48% of patients with poor platelet transfusion response. In this analysis that included only 27 patients, platelet response was measured 5.2 h after platelet transfusion and defined by the absence of platelet count increment [9]. In a large epidemiology study including a heterogeneous population of critically ill patients, Stanworth et al. reported no significant change of the mean platelet count pre- and post-transfusion [14]. In contrast to our findings, another study in mainly post-cardiac surgical patients found only one-fifth of platelet transfusions had poor platelet count increment [8]. In this study, patients were not significantly thrombocytopenic and patients with and without bleeding were also included. Although our study was conducted in nine ICUs (including medical, surgical and medico-surgical ICUs), the majority of the patients had haematological malignancies and chemotherapy. This reflects the higher need of prophylactic platelet transfusion in patients with bone marrow failure (either due to disease or treatment) and limits the generalizability of our findings to general ICU populations. In our study population, the methods used to measure platelet transfusion response and the timing of post-transfusion platelet count measurement might have contributed to our finding of a higher percentage of transfusions with poor response than the figures reported previously. There is no consensus on when to evaluate platelet transfusion response. Studies investigating platelet refractoriness recommend to check serial post transfusion platelet counts. However, platelet count is not routinely measured at 1 or 5 h after prophylactic platelet transfusion [15]. We found a good correlation between CCI and absolute platelet count increment. This is important as the CCI cannot be easily calculated in routine practice, as the body surface area and administered platelet dose might not be readily available for clinicians. The subgroup finding of very low platelet transfusion response in patients with haematology malignancy in ICU highlights the difference in this patient population and their potential specific requirements.

Our study identified factors independently associated with poor platelet increment. Sepsis as primary diagnosis had the strongest association with poor platelet response in the whole cohort and in subgroup analyses. We found that an increased storage duration negatively impacted on platelet transfusion response. An association between prolonged platelet storage duration and transfusion efficacy had been reported in haematology patients, but not in a general ICU population [7, 16]. We also observed that transfusion in patients with higher BMI seemed to protect against a poor transfusion response. A protective effect of BMI has been previously reported [7] and could be attributed to the higher platelet dose delivered in overweight patients in our study (since the platelet dose was calculated on the basis of body weight, whereas the blood volume of obese patients does not increase linearly with their weight).

In keeping with previous findings, we did not find any association between poor platelet transfusion response and mortality [7, 17, 18]. We did not find any difference in bleeding events nor in blood component requirements within the 24 h after platelet transfusion with and without optimal response.

Our study has some strengths; it is a prospective multicentre study including a heterogeneous critically ill population, supporting its external validity. The definition of poor platelet transfusion response is standardized based on the CCI that adjusts for platelet dose and body surface area. The duration between pre- and post-transfusion platelet count was consistent. We investigated risk factors for platelet transfusion response among patient-related parameters at admission and prior to transfusion and platelet-related parameters as well. We analysed clinical outcomes including mortality and bleeding within 24 h following transfusion using a well-established classification. However, our study suffers limitations including the observational design and a possible lack of power to draw any definitive conclusions on the association between platelet transfusion response and clinical outcomes, especially in subgroups’ analyses. Secondly, although the measurement of platelet count the day after transfusion is routine practice, a shorter time following transfusion may provide more accurate information regarding response, especially in patients with bone marrow failure [19]. Different CCI values to define poor platelet response have been used [12] [15]; however, the CCI value of 7 has been recommended by the French guidelines and was previously used in the critically ill population [6, 7]. Third, although this was a prospective study, we did have missing data for some variables. In order to maintain power and minimize bias, we imputed these missing data, which might have impacted on the results. Fourth, although the usefulness of platelet transfusion in some clinical settings may be questionable, the design of our study did not allow us to investigate this crucial issue. Randomized controlled trials would be required to properly assess the utility of this treatment. Fifth, in the study population, 77 patients received chemotherapy in the context of haematological malignancy, exposing them to the risk of bone marrow failure with an increased need for iterative platelet transfusion. Among these patients, assessment of platelet transfusion response might be done earlier; however, our study was a non-interventional study, and in most of critically ill patients with thrombocytopenia without bleeding, platelet count is only checked daily. Moreover, the high proportion of patients with haematological malignancies may limit the generalizability of our results to a general ICU population. Finally, although higher maximum storage duration of platelet concentrate was associated with suboptimal platelet transfusion response, we cannot draw any conclusion on whether prolonged storage duration is associated with worth patient clinical outcomes and what storage duration would be the most suitable.

## Conclusions

In conclusion, platelet transfusion response measured with the CCI at 18–24 h was suboptimal in more than three quarters of the study cohort, which included a majority of patients at risk of bone marrow failure. Among the parameters that were independently associated with poor platelet increment at 18–24 h, platelet storage duration was the only amenable to intervention.

### Supplementary Information

**Additional file 1. Supplemental Figure 1.** Receiver operating characteristic curves for platelet count increment and CCI. **Supplementary Table 1.** Details of surgery and invasive procedures requiring prophylactic platelet transfusion. **Supplementary Tables 2 to 8.** Sensitivity analyses in patients with and without hematology malignancy and chemotherapy.

## Data Availability

The data sets used and/or analysed during the current study are available from the corresponding author on reasonable request.

## Abbreviation

ICU: Intensive care unit
CCI: Corrected Count Increment
