# Supplementary material for: Prophylactic platelet transfusion response in critically ill patients: a prospective multicentre observational study
Source: Crit Care. 2023 Sep 27;27:373. doi: 10.1186/s13054-023-04650-z (PMC10537531; doi:10.1186/s13054-023-04650-z)
Supplement: Supplementary file 1 — Additional file 1. Supplemental Figure 1. Receiver operating characteristic curves for platelet count increment and CCI. Supplementary Table 1. Details of surgery and invasive procedures requiring prophylactic platelet transfusion. Supplementary Tables 2 to 8. Sensitivity analyses in patients with and without hematology malignancy and chemotherapy. [file 13054_2023_4650_MOESM1_ESM.docx]

**Supplemental Figure 1** Receiver operating characteristic curves for platelet count increment and CCI. Platelet count increment is plotted as a continuous variable

**
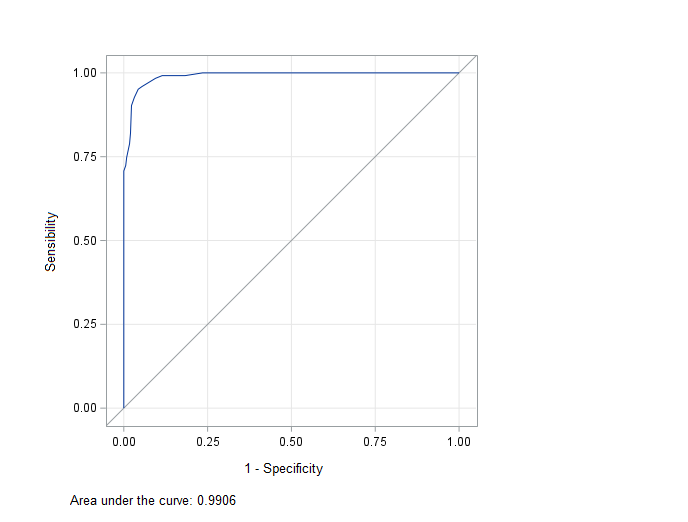
**

| **Platelet count increment (x10^9^/L)** | **Number of patients** | **False positive (n)** | **False negative (n)** | **Specificity** | **Sensibility** | **PPV** | **NPV** |
| --- | --- | --- | --- | --- | --- | --- | --- |
| 15 | 117 | 15 | 6 | 0.96 | 0.95 | 0.89 | 0.98 |
| 20 | 92 | 3 | 31 | 0.99 | 0.75 | 0.97 | 0.92 |
| 30 | 52 | 0 | 71 | 1.00 | 0.42 | 1.00 | 0.83 |

*PPV* positive predictive values, *NPV* negative predictive values

**Supplementary Table 1.** Details of surgery and invasive procedures requiring prophylactic platelet transfusion

| **Invasive procedures or surgery requiring platelet transfusion (n=163)** | (%) |
| --- | --- |
| Surgery | 52 (31.9) |
| Central venous catheter | 43 (26.4) |
| Lumbar puncture | 18 (11.0) |
| Endoscopy | 14 (8.6) |
| Pleural drain | 13 (8.0) |
| Biopsy | 6 (3.7) |
| Other | 17 (10.4) |

**Supplementary Table 2.** Baseline characteristics of patients with haematological malignancies and chemotherapy according to platelet transfusion response

| Variables | All patients  n=77 | Patients with at least one PT with poor response  n=72 | Patients without poor PT response  n=5 | *P* value |
| --- | --- | --- | --- | --- |
| **Demographic characteristics** | | | | |
| Age, years | 60 (46-66) | 60 (45-66) | 68 (60-72) | 0.0682 |
| Male sex | 43 (55.8%) | 40 (55.6%) | 3 (60.0%) | 1.0000 |
| Pregnancy^a^ | 22 (30.6%) | 21 (30.9%) | 1 (25.0%) | 1.0000 |
| BMI | 25.7 (22.2-28.4) | 25.6 (21.8-28.7) | 26.0 (23.1-27.4) | 0.9918 |
| History of platelet transfusion^b^ | 54 (85.7%) | 49 (84.5%) | 5 (100.0%) | 1.0000 |
| Cardiovascular disease | 12 (15.6%) | 11 (15.3%) | 1 (20.0%) | 0.5819 |
| Diabetes mellitus | 5 (6.5%) | 4 (5.6%) | 1 (20.0%) | 0.2918 |
| Solid Neoplasia | 5 (6.5%) | 5 (6.9%) | 0 (0.0%) | 1.0000 |
| Chronic respiratory disease | 6 (7.8%) | 6 (8.3%) | 0 (0.0%) | 1.0000 |
| ​​Chronic kidney injury | - | - | - | - |
| Anti-platelet agents | 6 (7.8%) | 5 (6.9%) | 1 (20.0%) | 0.3410 |
| Type of admission  Post-operative admission  Medical admission | 1 (1.3%)  76 (98.7%) | 1 (1.4%)  71 (98.6%) | 0 (0.0%)  5 (100.0%) | 1.0000 |
| Primary ICU admission diagnosis sepsis | 31 (40.3%) | 30 (41.7%) | 1 (20.0%) | 0.6427 |
| Diagnosis at ICU admission |  |  |  | 0.2108 |
| Respiratory distress | 21 (27.3%) | 20 (27.8%) | 1 (20.0%) |  |
| Neurologic | 4 (5.2%) | 3 (4.2%) | 1 (20.0%) |  |
| Cardiovascular | 1 (1.3%) | 1 (1.4%) | 0 (0.0%) |  |
| Hematologic | 2 (2.6%) | 2 (2.8%) | 0 (0.0%) |  |
| Hemorrhage | 8 (10.4%) | 7 (9.7%) | 1 (20.0%) |  |
| Metabolic | 3 (3.9%) | 2 (2.8%) | 1 (20.0%) |  |
| Other | 7 (9.1%) | 7 (9.7%) | 0 (0.0%) |  |
| *Patient ABO Group^c^* |  |  |  | 1.0000 |
| A | 37 (50.7%) | 35 (50.7%) | 2 (50.0%) |  |
| O | 27 (37.0%) | 25 (36.2%) | 2 (50.0%) |  |
| B | 7 (9.6%) | 7 (10.1%) | 0 (0.0%) |  |
| AB | 2 (2.7%) | 2 (2.9%) | 0 (0.0%) |  |
| Rhesus positive^d^ | 67 (91.8%) | 64 (92.8%) | 3 (75.0%) | 0.2958 |
| **Clinical and laboratory findings at ICU admission** | | | | |
| SAPS II | 49 (41-60) | 49 (41-62) | 45 (42-52) | 0.6210 |
| Platelet count, x 10^9^/L | 18 (9-29) | 18 (8-27) | 29 (19-38) | 0.1698 |
| Haemoglobin, g/dl | 7.9 (7.3-9.1) | 7.9 (7.3-9.0) | 8.2 (7.4-9.5) | 0.5922 |
| PT < 70% | 49 (69.0%) | 47 (71.2%) | 2 (40.0%) | 0.1687 |
| **Clinical outcomes** |  |  |  |  |
| Number of RBC units transfused | 0 (0-3) | 0 (0-3) | 0 (0-0) | 0.4037 |
| ICU LOS, days | 7 (3-15) | 7 (3-16) | 3 (2-3) | 0.0397 |
| Hospital LOS, days | 25 (11-38) | 25 (11-38) | 18 (9-29) | 0.4905 |
| MV duration, days | 0 (0-8) | 0 (0-8) | 0 (0-0) | 0.0582 |
| Survival at ICU discharge | 57 (74.0%) | 52 (72.2%) | 5 (100.0%) | 0.3188 |

Data are presented as median (IQR: interquartile) or n (%).

*Abbreviations*: *BMI* Body Mass Index, *ICU* Intensive Care Unit, *LOS* Length of stay, *RBC* Red Blood Cells, *PT* Prothrombin Time, *SAPS II* Simplified Acute Physiology Score II, *MV* mechanical ventilation

^a^ Missing data: n=5; ^b^ Missing data: n=14; ^c^ Missing data: n=4; ^d^ Missing datas: n=4; ^e^ Missing data: n=6

**Supplementary Table 3.** Comparison of characteristics of platelet transfusion with and without suboptimal response based on a CCI < 7 in patients with haematological malignancies and chemotherapy

| Variables | All episodes  n=268 | Suboptimal PT response  n=212 | Optimal PT response  n=56 | *P* value |
| --- | --- | --- | --- | --- |
| **Clinical and biological features before transfusion** | | | | |
| Heart rate (/min) | 105 (94-120) | 106 (95-120) | 100 (90-114) | 0.0479 |
| Heart rate >100/min | 175 (65.3%) | 144 (67.9%) | 31 (55.4%) | 0.0789 |
| Temperature, °C^a^ | 37.1 (36.5-37.8) | 37.1 (36.5-37.8) | 37.1 (36.5-37.8) | 0.7922 |
| Temperature>39°C^a^ | 16 (6.2%) | 14 (6.8%) | 2 (3.6%) | 0.5350 |
| Mean arterial BP, mmHg | 75 (68-87) | 75 (68-87) | 76 (68-84) | 0.9321 |
| Therapeutic anticoagulation | 17 (6.3%) | 11 (5.2%) | 6 (10.7%) | 0.1338 |
| AKI requiring RRT | 28 (10.4%) | 22 (10.4%) | 6 (10.7%) | 0.9416 |
| ECMO | 7 (2.6%) | 5 (2.4%) | 2 (3.6%) | 0.6389 |
| Infection requiring antibiotics | 238 (88.8%) | 187 (88.2%) | 51 (91.1%) | 0.5455 |
| SOFA score^b^ | 7 (6-11) | 8 (6-12) | 6 (5-8) | 0.0009 |
| Platelet count, x 10^9^/L | 14 (8-21) | 13 (8-20) | 16 (9-26) | 0.0266 |
| **Transfusion related characteristics** | |  |  |  |
| *Transfusion Indication* |  |  |  | 0.9536 |
| Prophylactic without invasive procedure | 63 (23.5%) | 50 (23.6%) | 13 (23.2%) |  |
| Prophylactic prior Invasive procedure | 205 (76.5%) | 162 (76.4%) | 43 (76.8%) |  |
| Platelet dose | 3.8 (3.3-4.7) | 3.7 (3.3-4.7) | 3.9 (3.3-4.8) | 0.7425 |
| Apheresis platelet concentrate | 101 (37.7%) | 77 (36.3%) | 24 (42.9%) | 0.3693 |
| Pooled platelet concentrate | 167 (62.3%) | 135 (63.7%) | 32 (57.1%) |  |
| ABO compatibility^c^ | 199 (78.3%) | 155 (77.1%) | 44 (83.0%) | 0.3532 |
| Maximum platelet storage duration >3 days | 4 (3-5) | 4 (3-5) | 4 (3-5) | 0.1849 |
| 24 hours fluid balance, mL^d^ | 700 (-624-1727) | 658 (-629-1726) | 722 (-500-1783) | 0.9838 |

Data are presented as median (IQR: interquartile), n (%). P values comparing patients are tested by Mann-Whitney (continuous variables) and Chi2 or Fisher tests (categorical variables).

*Abbreviations*: *AKI* Acute Kidney Injury, *BP* Blood Pressure, *ECMO* Extracorporeal Membrane Oxygenation, *RRT* Renal Replacement Therapy, *SOFA* Sequential Organ Failure Assessment

^a^ Missing data: n=8; ^b^ Missing data: n=19; ^c^ Missing data: n=14; ^d^ Missing data: n=9

**Supplementary Table 4.** Univariate and multivariate mixed effect model to evaluate association between demographic and transfusion related characteristics with poor platelet transfusion response in patients with haematological malignancies and chemotherapy

|  | Univariate analysis | | | |  | | Multivariate analysis | | | |
| --- | --- | --- | --- | --- | --- | --- | --- | --- | --- | --- |
| Variables | OR | 95% CI | *P* value | |  | OR | | | 95% CI | *P* value |
| **Baseline characteristics** | | | | |  | |  |  | |  |
| Age (years) | 0.99 | [0.96-1.01] | | 0.2500 |  | |  |  | |  |
| Gender (ref: male sex) | 1.28 | [0.56-2.93] | | 0.5529 |  | |  |  | |  |
| BMI | 0.96 | [0.90-1.03] | | 0.2507 |  | |  |  | |  |
| Pregnancy | 1.76 | [0.71-4.39] | | 0.2233 |  | |  |  | |  |
| Cardiovascular disease | 1.85 | [0.52-6.61] | | 0.3400 |  | |  |  | |  |
| SAPS II | 1.01 | [0.98-1.03] | | 0.5937 |  | |  |  | |  |
| Antecedent of platelet transfusion (ref="No") | 1.18 | [0.43-3.28] | | 0.7487 |  | |  |  | |  |
| Platelet count at ICU admission | 0.96 | [0.93-1.00] | | 0.0297 |  | |  |  | |  |
| Haemoglobin | 0.88 | [0.65-1.19] | | 0.4180 |  | |  |  | |  |
| PT (ref="<70%") | 0.52 | [0.23-1.21] | | 0.1281 |  | |  |  | |  |
| Primary ICU admission diagnosis sepsis (ref:No) | 2.84 | [1.22-6.60] | | 0.0157 |  | | 2.43 | [1.05-5.64] | | 0.390 |
| **Transfusion episodes related features** | | | | |  | |  |  | |  |
| Heart rate before transfusion >100/min | 1.64 | [0.85-3.17] | | 0.1380 |  | |  |  | |  |
| Temperature before transfusion (ref <39°C) | 2.19 | [0.44-11.0] | | 0.3416 |  | |  |  | |  |
| Infection requiring antibiotics | 0.69 | [0.23-2.10] | | 0.5142 |  | |  |  | |  |
| Systolic arterial blood pressure before transfusion | 1.00 | [0.98-1.01] | | 0.5084 |  | |  |  | |  |
| Therapeutic anticoagulation | 0.67 | [0.17-2.65] | | 0.5627 |  | |  |  | |  |
| SOFA score | 1.12 | [1.02-1.23] | | 0.0192 |  | | 1.1 | [1.00-1.21] | | 0.0472 |
| 24 hours fluid balance, mL | 1.00 | [1.00-1.00] | | 0.7724 |  | |  |  | |  |
| Maximum platelet storage duration | 1.19 | [0.91-1.57] | | 0.2064 |  | |  |  | |  |

The corrected count increment was treated as a binary variable (upper than 7 or not). A positive estimate means that an increase in the variable of interest is associated with a platelet transfusion with a CCI>7, whereas a negative one is the opposite.

*Abbreviations*: *BMI* Body Mass Index, *PT* Prothrombin Time, *SAPS II* Simplified Acute Physiology Score II, *SOFA* Sequential Organ Failure Assessment

**Supplementary Table 5.** Bleeding events and transfusion within 24 hours after platelet transfusion according to platelet transfusion response in patients with haematological malignancies and chemotherapy

| Variables | | All episodes  n=268 | Suboptimal PT response  n=212 | Optimal PT response  n=56 | *P* value |
| --- | --- | --- | --- | --- | --- |
| *WHO Grade 2 to 4 bleeding* |  | |  |  |  |
| Indication for transfusion:  Thrombocytopenia with surgery or invasive procedure (n=63) | 4 (6.3%) | | 4 (8.0%) | 0 (0.0%) | 0.5722 |
| Thrombocytopenia without surgery or invasive procedure (n=205) | 27 (13.2%) | | 23 (14.2%) | 4 (9.3%) | 0.6116 |
| *WHO Grade 3 or 4 bleeding* |  | |  |  |  |
| Indication for transfusion:  Thrombocytopenia with surgery or invasive procedure (n=4) | 2 (50.0%) | | 2 (50.0%) | 0 (0.0%) | 1.000 |
| Thrombocytopenia without surgery or invasive procedure (n=27) | 20 (74.1%) | | 18 (78.3%) | 2 (50%) | 0.5546 |
| *Transfusions within the 24 hours after platelet transfusion* |  | |  |  |  |
| RBC units | 2.0 (0-12) | | 2 (0-10) | 6 (0-14) | 0.0396 |
| Requirement of at least one RBC unit | 146 (55.2%) | | 109 (52.2%) | 37 (66.1%) | 0.0629 |
| Requirement of at least one FFP | 33 (12.3%) | | 27 (12.7%) | 6 (10.7%) | 0.6573 |

Data are presented as median (IQR: interquartile), n (%). P values comparing patients are tested by Mann-Whitney (continuous variables) and Chi2 or Fisher tests (categorical variables).

*Abbreviations*: *FFP* Fresh Frozen Plasma, *PT* Platelet Transfusion, *RBC* Red Blood Cells, *WHO* World Health Organization

**Supplementary Table 6.** Baseline characteristics of patients (excluding patients with haematological malignancies and chemotherapy) according to platelet transfusion with poor response

| Variables | All patients  n=104 | Patients with at least one PT with poor response  n=69 | Patients without poor PT response  n=35 | *P* value |
| --- | --- | --- | --- | --- |
| **Demographic characteristics** | | | | |
| Age, years | 61 (49-70) | 59 (47-68) | 66 (53-73) | 0.1046 |
| Male sex | 60 (57.7%) | 37 (53.6%) | 23 (65.7%) | 0.2383 |
| Pregnancy^a^ | 33 (35.1%) | 25 (40.3%) | 8 (25.0%) | 0.1403 |
| BMI | 24.7 (22.9-28.2) | 24.6 (22.6-28.1) | 24.7 (23.4-28.7) | 0.5646 |
| History of platelet transfusion^b^ | 22 (39.3%) | 15 (37.5%) | 7 (43.8%) | 0.6653 |
| Cardiovascular disease | 36 (34.6%) | 24 (34.8%) | 12 (34.3%) | 0.9599 |
| Diabetes mellitus | 16 (15.4%) | 11 (15.9%) | 5 (14.3%) | 0.8249 |
| Solid Neoplasia | 27 (26.0%) | 21 (30.4%) | 6 (17.1%) | 0.1440 |
| Chronic respiratory disease | 12 (11.5%) | 8 (11.6%) | 4 (11.4%) | 1.0000 |
| ​​Chronic kidney injury | 7 (6.7%) | 3 (4.3%) | 4 (11.4%) | 0.2214 |
| Anti-platelet agents | 18 (17.3%) | 11 (15.9%) | 7 (20.0%) | 0.6052 |
| Type of admission  Post-operative admission  Medical admission | 32 (15.4%)  176 (84.6%) | 22 (13.3%)  144 (86.7%) | 10 (23.8%)  32 (76.2%) |  |
| Post-operative admission | 22 (21.2%) | 13 (18.8%) | 9 (25.7%) | 0.4173 |
| Medical admission | 82 (78.8%) | 56 (81.2%) | 26 (74.3%) |  |
| Primary ICU admission diagnosis sepsis | 35 (33.7%) | 29 (42.0%) | 6 (17.1%) | 0.0112 |
| Diagnosis at ICU admission |  |  |  | 0.1362 |
| Respiratory distress | 8 (7.7%) | 4 (5.8%) | 4 (11.4%) |  |
| Trauma | 4 (3.9%) | 1 (1.4%) | 3 (8.6%) |  |
| Neurologic | 7 (6.7%) | 5 (7.2%) | 2 (5.7%) |  |
| Cardiovascular | 6 (5.8%) | 3 (4.3%) | 3 (8.6%) |  |
| Hematologic | 7 (6.7%) | 5 (7.2%) | 2 (5.7%) |  |
| Uro-digestive | 3 (2.9%) | 2 (2.9%) | 1 (2.9%) |  |
| Hemorrhage | 9 (8.7%) | 5 (7.2%) | 4 (11.4%) |  |
| Metabolic | 3 (2.9%) | 2 (2.9%) | 1 (2.9%) |  |
| Other | 22 (21.2%) | 13 (18.8%) | 9 (25.7%) |  |
| *Patient ABO Group^c^* |  |  |  | 0.8172 |
| A | 36 (36.7%) | 25 (37.9%) | 11 (34.4%) |  |
| O | 46 (46.9%) | 29 (43.9%) | 17 (53.1%) |  |
| B | 14 (14.3%) | 10 (15.2%) | 4 (12.5%) |  |
| AB | 2 (2.0%) | 2 (3.0%) | 0 (0.0%) |  |
| Rhesus positive^d^ | 83 (84.7%) | 56 (83.6%) | 27 (87.1%) | 0.7694 |
| **Clinical and laboratory findings at ICU admission** | | | | |
| SAPS II^e^ | 54 (40-68) | 55 (41-71) | 52 (35-65) | 0.2920 |
| Platelet count, x 10^9^/L | 57 (30-108) | 53 (28-95) | 64 (38-156) | 0.1516 |
| Haemoglobin, g/dl | 10.0 (8.2-12.3) | 9.2 (7.7-11.1) | 11.9 (9.6-13.5) | 0.0010 |
| PT < 70%^f^ | 73 (72.3%) | 50 (75.8%) | 23 (65.7%) | 0.2833 |
| **Clinical outcomes** |  |  |  |  |
| Number of RBC units transfused | 0 (0-3) | 0 (0-3) | 0 (0-3) | 0.6163 |
| ICU LOS, days | 12 (6-23) | 13 (6-24) | 12 (7-23) | 0.7159 |
| Hospital LOS, days | 23 (12-47) | 23 (12-60) | 26 (12-39) | 0.3291 |
| MV duration, days | 6 (0-13) | 7 (0-14) | 6 (1-12) | 0.8323 |
| Survival at ICU discharge | 76 (73.1%) | 52 (75.4%) | 24 (68.6%) | 0.4607 |

^a^ Missing values: n=10; ^b^ Missing values: n=48; ^c^ Missing values: n=6; ^d^ Missing values: n=6; ^e^ Missing values: n=1; ^f^ Missing values: n=3

Data are presented as median (IQR: interquartile) or n (%).

*Abbreviations*: *BMI* Body Mass Index, *ICU* Intensive Care Unit, *LOS* Length of stay, *RBC* Red Blood Cells, *PT* Prothrombin Time, *SAPS II* Simplified Acute Physiology Score II, *MV* mechanical ventilation

**Supplementary Table 7.** Comparison of characteristics of platelet transfusion with and without suboptimal response based on a CCI < 7 (excluding patients with haematological malignancies and chemotherapy)

| Variables | All episodes  n=204 | Suboptimal PT response  n=137 | Optimal PT response  n=67 | *P* value |
| --- | --- | --- | --- | --- |
| **Clinical and biological features before transfusion** | | | | |
| Heart rate (/min) | 100 (85-116) | 105 (86-120) | 92 (85-108) | 0.0041 |
| Heart rate >100/min | 107 (52.5%) | 80 (58.4%) | 27 (40.3%) | 0.0151 |
| Temperature, °C^a^ | 36.8 (36.3-37.4) | 37.0 (36.3-37.5) | 36.7 (36.3-37.2) | 0.0629 |
| Temperature>39°C^a^ | 4 (2.0%) | 4 (3.0%) | 0 (0.0%) | 0.3050 |
| Mean arterial BP, mmHg | 78 (71-89) | 77 (70-92) | 79 (72-85) | 0.8717 |
| Therapeutic anticoagulation | 33 (16.2%) | 17 (12.4%) | 16 (23.9%) | 0.0366 |
| AKI requiring RRT | 69 (33.8%) | 42 (30.7%) | 27 (40.3%) | 0.1716 |
| ECMO | 1 (0.5%) | 1 (0.7%) | 0 (0.0%) | 1.0000 |
| Infection requiring antibiotics | 159 (77.9%) | 111 (81.0%) | 48 (71.6%) | 0.1292 |
| SOFA score^b^ | 10 (7-14) | 11 (7-15) | 10 (7-14) | 0.3740 |
| Platelet count, x 10^9^/L | 19 (11-33) | 17 (9-31) | 22 (15-43) | 0.0068 |
| **Transfusion related characteristics** | |  |  |  |
| *Transfusion Indication* |  |  |  | 0.1241 |
| Prophylactic without invasive procedure | 100 (49.0%) | 62 (45.3%) | 38 (56.7%) |  |
| Prophylactic prior Invasive procedure | 104 (51.0%) | 75 (54.7%) | 29 (43.3%) |  |
| Platelet dose | 3.6 (3.3-4.8) | 3.8 (3.3-4.8) | 3.6 (3.3-4.8) | 0.6259 |
| Apheresis platelet concentrate | 54 (26.5%) | 42 (30.7%) | 12 (17.9%) | 0.0526 |
| Pooled platelet concentrate | 150 (73.5%) | 95 (69.3%) | 55 (82.1%) |  |
| ABO compatibility^c^ | 148 (76.7%) | 101 (76.5%) | 47 (77.0%) | 0.9350 |
| Maximum platelet storage | 4 (4-5) | 5 (4-5) | 4 (3-5) | 0.0151 |
| 24 hours fluid balance, mL^d^ | 1000 (9-2078) | 1000 (105-2150) | 1027 (9-2039) | 0.8601 |

Data are presented as median (IQR: interquartile), n (%). P values comparing patients are tested by Mann-Whitney (continuous variables) and Chi2 or Fisher tests (categorical variables).

*Abbreviations*: *AKI* Acute Kidney Injury, *BP* Blood Pressure, *ECMO* Extracorporeal Membrane Oxygenation, *RRT* Renal Replacement Therapy, *SOFA* Sequential Organ Failure Assessment

^a^ Missing data n= 6; ^b^ Missing data n= 4; ^c^ Missing data n= 11; ^d^ Missing data n= 5

**Supplementary Table 8.** Univariate and multivariate mixed effect model to evaluate association between demographic and transfusion related characteristics with poor platelet transfusion response (excluding patients with haematological malignancies and chemotherapy)

|  | Univariate Analysis | | |  | | Multivariate analysis | | | |
| --- | --- | --- | --- | --- | --- | --- | --- | --- | --- |
| Variables | OR | 95% CI | *P* value |  | Estimate | | | 95% CI | *P* value |
| **Baseline characteristics** | | | |  | |  |  | |  |
| Age (years) | 0.99 | [0.97-1.02] | 0.5731 |  | |  |  | |  |
| Gender (ref: male sex) | 1.37 | [0.68- 2.74] | 0.3746 |  | |  |  | |  |
| BMI | 0.96 | [0.91- 1.02] | 0.2299 |  | |  |  | |  |
| Pregnancy | 1.48 | [0.73-2.97] | 0.2766 |  | |  |  | |  |
| Cardiovascular disease | 1.03 | [0.49-2.18] | 0.9280 |  | |  |  | |  |
| Chronic kidney injury | 0.31 | [0.07-1.34] | 0.1164 |  | |  |  | |  |
| SAPS II | 1.01 | [0.99-1.02] | 0.5110 |  | |  |  | |  |
| Antecedent of platelet transfusion (ref="No") | 0.45 | [0.19-1.05] | 0.0645 |  | |  |  | |  |
| Platelet count at ICU admission | 1.00 | [0.99-1.01] | 0.5550 |  | |  |  | |  |
| Haemoglobin | 0.82 | [0.72-0.93] | 0.0022 |  | | 0.81 | [0.71-0.93] | | 0.0022 |
| PT (ref="<70%") | 0.84 | [0.39-1.84] | 0.6687 |  | |  |  | |  |
| Type of admission (ref=postoperative) | 2.04 | [0.91-4.60] | 0.0832 |  | |  |  | |  |
| Primary ICU admission diagnosis sepsis (ref:No) | 2.83 | [1.31-6.11] | 0.0085 |  | | 2.82 | [1.25-6.35] | | 0.0130 |
| **Transfusion episodes related features** | | | |  | |  |  | |  |
| Heart rate before transfusion >100/min | 2.00 | [1.05-3.78] | 0.0342 |  | |  |  | |  |
| Infection requiring antibiotics | 1.71 | [0.81-3.63] | 0.1603 |  | |  |  | |  |
| Systolic arterial blood pressure before transfusion | 1.00 | [0.99-1.02] | 0.7830 |  | |  |  | |  |
| Therapeutic anticoagulation | 0.48 | [0.21-1.11] | 0.0850 |  | |  |  | |  |
| SOFA score | 1.04 | [0.97-1.12] | 0.2414 |  | |  |  | |  |
| 24 hours fluid balance | 1.00 | [1.00-1.00] | 0.9107 |  | |  |  | |  |
| Maximum platelet storage duration | 1.43 | [1.07-1.90] | 0.0147 |  | | 1.37 | [1.02-1.85] | | 0.0371 |

The corrected count increment was treated as a binary variable (upper than 7 or not). A positive estimate means that an increase in the variable of interest is associated with a platelet transfusion with a CCI>7, whereas a negative one is the opposite.

*Abbreviations*: *BMI* Body Mass Index, *PT* Prothrombin Time, *SAPS II* Simplified Acute Physiology Score II, *SOFA* Sequential Organ Failure Assessment
